# Mediation effects of cognitive, physical, and motivational reserves on cognitive performance in older people

**DOI:** 10.3389/fpsyg.2022.1112308

**Published:** 2023-01-17

**Authors:** Antonio Sánchez Cabaco, Marina Wobbeking Sánchez, Manuel Mejía-Ramírez, José David Urchaga-Litago, Eduardo Castillo-Riedel, Beatriz Bonete-López

**Affiliations:** ^1^Faculty of Psychology, Pontifical University of Salamanca, Salamanca, Spain; ^2^Department of Psychology, Pontificial University of Salamanca, Salamanca, Spain; ^3^School of Psychology, CETYS University, Tijuana, Mexico; ^4^Faculty of Communications, Pontifical University of Salamanca, Salamanca, Spain; ^5^Department of Health Psychology, University of Miguel Hernández de Elche, Elche, Spain

**Keywords:** cognitive reserve, physical reserve, motivational reserve, cognitive impairment, age, educational level

## Abstract

**Introduction:**

We study from a multidimensional perspective the different factors that help prevent the development of cognitive impairment in old aging.

**Methods:**

This study analyzed in 300 elderly subjects the relationship between cognitive reserve (CR), physical reserve (PR) and motivational reserve (MR) with cognitive impairment. This study also takes into consideration different variables (sex, age, educational level, and institutionalization) that might affect the results in the different types of reserves (CR, physical and MR) and cognitive impairment.

**Results:**

The results show that people with a higher cognitive reserve, physical reserve and motivational reserve have less cognitive impairment.

**Discussion:**

Therefore, it is important to consider measuring the CR as a variable to diagnose neurodegenerative illnesses but it is also essential to consider the physical state and physical activity, as well as the motivational dimension. With the cognitive reserve and sex variables no significant differences were observed. Age had a negative effect on strategic flexibility, but those with higher CR had better cognitive flexibility and the educational.

## Introduction

The increase in life expectancy all over the world has proven that the older person’s demographic continues to grow with time (Jiménez et al., 2021). The World Health Organization (World Health Organization, 2021) states that people are living longer than ever before, this is something that is being experienced all over the world, not only has the quantity of older people increased but also the proportion of older people in relation to the general population. This will become more evident between the years 2020 and 2050, when it is estimated that the number of people aged 80 and over will triple in size until it reaches 426 million in population. Aging occurs as a result of high accumulation of cellular and molecular damage over the years, which produces a progressive decrease in physical and mental capabilities, increasing the risk of developing an illness and eventually death. Although these changes occur with aging, they are not uniform nor linear, and their relation with a person’s age is quite relative (World Health Organization, 2021). In this sense, as Barba (2021) stated, due to the increase in life expectancy, there has been a significant increase in age-related diseases, both physical and neuropsychological. Therefore, due to these demographic changes it is imperative to be prepared and attend aging in a multidisciplinary manner (Ochoa-Vázquez et al., 2019).

Currently there are various indicators that have a protective role in the presence of cognitive impairment, that favors a healthy and active aging process. Firstly, the cognitive reserve (CR) defined by Stern (2002) as the organism’s ability to resist brain deterioration without presenting symptoms. According to this variable, people with a higher cognitive reserve have a lower risk and vulnerability of suffering a degenerative pathological process in cognitive impairment in old age. The current research based on active CR models suggests that there are several variables that influence its development, maintenance and enhancement throughout life. Although currently there is no existing evidence that suggests the relevance of each of the components, as well as the most appropriate combination of the measure, for its operationalization variables such as employment, education and leisure activities have been used: physical, mental and social, which have been the most used obtaining conclusive results (Barba, 2021).

The cognitive reserve hypothesis tries to explain the individual differences in regard to the vulnerability due to brain changes related to age or illness (Stern, 2005). It is thought that positive influences throughout life (better education or higher level of literacy) increase the efficacy of cognitive processes in aging (Stern, 2009). Although there is much literature on cognitive cues, there is not much evidence on the influence of emotional or motivational cues in delaying cognitive impairment. Thus, in a recent work by Guerrini and her team (Guerrini et al., 2022), they address the relation of cognitive reserve as being a key in emotion recognition, that is, the ability to interpret and combine social cues within different sensory systems. The results show the importance of cognitive reserve in executive functioning (assessed through Stroop test) but not in improving scores related to emotional performance (emotional recognition through faces or voices). Therefore, it would seem that in light of these results, cognitive reserve indicators are good predictors of cognitive task performance, although not being as effective in social cognition tests.

The cognitive reserve model that underlines the approach of this research refers to the dimension of resilience in the presence of damage or pathology, and also to cognitive decline or deterioration associated with age (Stern and Barulli, 2019). It is understood that the dichotomy between cognitive and cerebral reserve is not defensible given the empirical evidence of the close relationship between the two from the neurophysiological level (Barulli and Stern, 2013). In the same way, the differences are not shared with other constructs such as brain maintenance (Opdebeeck et al., 2016), because they are based on similar key aspects (education, cognitive stimulation or social contact). The operationalization of our proposal broadens the initial horizon of cognitive reserve (Stern, 2002) because it is supported by the high correlation between cognitive reserve and environmental enrichment, resulting in physical and mental activity as moderator variables of brain deterioration (Nithianantharajah and Hannan, 2009). The cognitive reserve construct that integrates the most empirically validated formulation refers to a combination of resistance capacity (brain processes in the face of pathology) and resilience (coping in terms of cognitive performance), which would integrate the concepts of cognitive reserve and brain maintenance, previously separated artificially (Arenaza-Urquijo and Vemuri, 2018). In this explanatory framework of cognitive reserve as a resilient mechanism, the approach can be understood associating it with physical and motivational reserves. There is more evidence of the association with the physical reserve (Cheng, 2016), while studies that relate to motivational keys of meaning are more recent (Bartrés-Faz et al., 2018), and scarce in its three-dimensional vision (Wobbeking et al., 2021).

Secondly, physical activity in elderly people is a key and favorable element of a healthy lifestyle, since both exercise and sport are effective in preventing, treating and recovering from the presence of certain diseases (Aldas et al., 2021). Doing exercise benefits people in their physical and mental development, by being a factor that protects, promotes and maintains good health, quality of life and wellbeing. The World Health Organization, (2020) defines physical activity as the body movements generated by skeletal muscles that consume energy. Therefore, physical activity refers to all movements to or from a particular place or as part of work, or even during your leisure time.

Physical exercise has a positive effect on most of the physical and psycho-social functions of the older people. For this reason, regular physical exercise adapted for the older population is the best non-pharmacological therapy against the main illnesses associated with the aging process (Martínez et al., 2021). Not only does physical activity benefit physical aspects, but also emotional ones like self-esteem, it decreases depressive symptoms, it improves social relationships and helps delay cognitive decline. Due to all these elements, carrying out physical activity makes it possible for the person to decrease the moments of sedentary lifestyle since it is considered one of the greatest risk factors for developing heart disease and death, since evidence shows there is a direct relation between a sedentary lifestyle, physical inactivity and cardiovascular mortality (Rodríguez et al., 2020). These data coincide with those carried out in a bibliographic review in the last year by Martínez et al. (2021). The results show that daily practice of physical activity improves self-esteem and has a positive effect on happiness, in addition to favoring the capacity for self-care, improving the integration of the body scheme and facilitating intergenerational relationships.

Lastly, the motivational reserve (MR) stands out. Human motivation explains why people behave in a certain way. It involves affective, cognitive and learning components (Petri and Govern, 2021). A person with a high intrinsic motivation behaves in a direct, consistent and stable manner (Deci and Ryan, 1985). In older people, the meaning of life is especially important as a motivational factor (Pinquart, 2002). In addition, we also have to consider the positive effects and all the human strengths as part of the motivational aspects (Reich et al., 2004) that have been appropriate and developed, among which is worth highlighting the values and vital goals (Park et al., 2004; Peterson and Seligman, 2004). In older people, numerous studies have shown that having a better perspective on one’s own meaning of life is related to better psychosocial health, physical well-being, a lower risk of mortality and depression, less loneliness, more optimism and more allostatic load (Zilioli et al., 2015; Cohen et al., 2016; Steptoe and Fancourt, 2019; Kim et al., 2020, 2022).

The meaning of life is positively correlated with character strengths, which are also related to a better life satisfaction and happiness, better physical health and lower anxiety and depression (Kim et al., 2013; Proyer et al., 2013; Niemiec, 2014; Steptoe, 2019; Trudel-Fitzgerald et al., 2019; Weziak-Bialowolska et al., 2021).

Therefore, the motivational reserve built throughout life has been found to influence the psychological well-being and quality of life in older people, which include CR and physical well-being.

**Figure 1:**
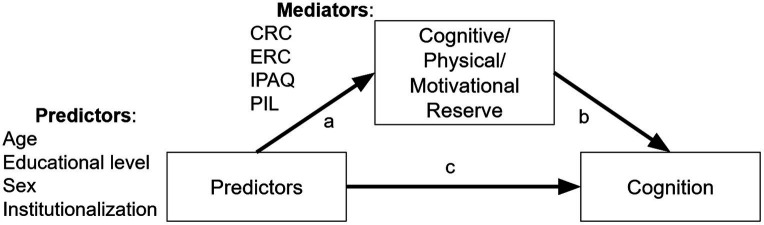
Structure of the mediation analyses tested. For each predictor (sociodemographic variables: age, education level, sex, institutionalization), we tested if their effect on cognition (measured with MoCA and M@T) was mediated by cognitive (CRQ, CRS scores), physical (IPAQ scores) and motivational (PIL scores) reserves. MoCA, Montreal Cognitive Assessment; M@T, Memory Alteration Test; CRQ, Cognitive Reserve Questionnaire; CRS, Cognitive Reserve Scale; IPAQ, International Physical Activity Questionnaire; PIL, Purpose in Life questionnaire.

The originality provided by the main objective of this study is to separately analyze the three components that in the cognitive reserve literature show controversies in the results and relation between: cognitive dimensions, physical activity and motivational cues. This research is a continuation of a previous study (Wobbeking et al., 2021) in which components associated with some types of reserve (physical and motivational) have been operationalized through a model of structural equations. This study provides evidence of the mediation effects of four sociodemographic predictors (age, educational level, sex and institutionalization) of cognitive performance contrasted with the three types of reserve (cognitive, physical and motivational) as mediating variables. This proposed model (Figure 1) hypothesizes that cognitive, physical, and motivational reserves are predictors of cognitive decline, as are the sociodemographic variables age, educational level, gender, and institutionalization. Cognitive impairment is the dependent variable in this model.

## Materials and methods

### Participants

The total sample consisted of 300 subjects: 224 women (75%) and 76 men (25%), in which 150 subjects live in institutionalized centers and 150 subjects live autonomously. The total sample consisted of subjects between the ages of 55 and 99 years, with a mean age of 74.66 for men and 74.70 for women, with a total mean age of 74.68 years. In regard to the institutionalized subjects, there were 107 women (71%) and 43 men (29%), between the ages of 55 years and 99 years, and whose mean age is 83.17 years. The subjects that were not institutionalized comprised a total of 117 women (78%) and 33 men (22%) between the ages of 55 years and 84 years, with a mean age of 66.21 years. The educational level for the total sample, 10% of the subjects have not had any type of education, being very close to illiteracy, 51% had primary education, 20% had secondary education and 19% had a higher education.

The inclusion criteria used were the same for both, firstly, being 55 years or older, having no symptoms of cognitive impairment and being institutionalized in a residential center or living at home.

### Instruments

The evaluation battery used has been a sociodemographic data sheet that includes information on the older people, such as sex, age, educational level and institutionalized center, if they belong to the subsample. And the battery of tests with the following questionnaires are answered without a time limit: *Cognitive Reserve Questionnaire* (*CRQ*; Rami et al., 2011). This test evaluates the cognitive reserve through a series of variables (8 variables), like the subjects educational level, training courses, the education of the parents and work occupation, musical training and language proficiency. It also measures the frequency of stimulating activities carried throughout life such as intellectual games and reading. The study shows an acceptable reliability (Cronbach’s Alpha: 0.72) and there is evidence of its validity.

*Cognitive Reserve Scale* (*CRS*; León et al., 2015). It is used to estimate the cognitive reserve in the Spanish population. It has a good reliability index (Cronbach’s Alpha: 0.80) and evidence of good validity (León et al., 2017). This scale consists of 24 items and is divided into four areas: activities of daily living, training and information, hobbies and interests, and social life.

*Montreal Cognitive Assessment* (*MoCA*; Nasreddine et al., 2005). This test was developed as a brief screening tool to detect mild cognitive impairment. It measures various cognitive domains such as visuospatial abilities, memory, language and attention. This scale consists of 13 subtests. The approximate time to administer the MoCa test is 15 min and the maximum score possible is 30 points, with a cutoff score equal to 26 points or above to signify absence of cognitive impairment. For Spain’s older adults population, this test has shown a good reliability (Cronbach’s Alpha: 0.77) and there is evidence of its validity (Lozano et al., 2019).

*Memory Alteration Test* (*M@T*; Rami et al., 2007). This is a validated screening test for amnesic-type mild cognitive impairment and for mild Alzheimer’s disease. This global memory screening test is made up of five subtests: encoding, temporal orientation, semantic memory, free recall and facilitated recall. This test has 38 items and shows an excellent reliability (Cronbach’s Alpha: 0.92) and there is evidence of its validity. The maximum score possible is 50 points and the approximate time to administer the test in healthy subjects is 10 min. This test has been validated by numerous studies (Rami, 2009; Ozer et al., 2016) and in other countries like Portugal (Sousa, 2015). This test has a sensitivity of 96% in the 37 cutoff points, and a specificity of 70% to differentiate between subjects with mild cognitive impairment with subjective memory complaints.

*International Physical Activity Questionnaire (IPAQ)*. This questionnaire was developed by World Health Organization (2012) and is made up of seven items in which the responses are quantified by the number of minutes or hours of activity practice, as indicated by each item. The reliability of the IPAQ in its short version (9 items) is 0.65 (rs = 0.76;CI95%:0.73–0.77; Craig et al., 2003). This scale helps obtain data related to physical activity associated with health and has currently been used with older people.

*Purpose In Life (PIL).* An attitude test designed by Crumbaugh and Maholick (1964) (Martínez et al., 2012) to quantify the degree to which the individual experiences that his life has meaning and purpose, as well as existential emptiness. This test is currently the most widely used instrument in research on the meaning of life due to the high internal consistency in all cases greater than 0.80 Cronbach’s Alpha obtained in numerous studies with different populations (Cohen et al., 2016). This test consists of 20 items, in which the subjects must individually answer questions using a Likert-type scale from 1 to 7 between two extreme feelings. The highest score possible for the PIL is 140, for its interpretation the following criteria have been used (Crumbaugh and Maholick, 1969): scores below 90 would mean a clear lack of meaning, scores between 90 and 105 would show a lack of definition regarding the meaning of life, while scores above 105 would indicate the clear presence of meaning in life. This criteria is used in general for the entire Spanish population. Therefore, the higher the score, the greater the meaning of life.

### Procedure

For this research, two stages were established. First, the centers were contacted in order to recruit the sample, both from the self-employed and institutionalized categories. The first group (non-institutionalized subjects) consisted of students from Experience University, Pontifical University of Salamanca and the SABIEX Program of the University of Miguel Hernández de Elche. These university programs for seniors are based on lifelong learning models. These training programs for the elderly are carried out in the university context, and are established in all the universities in Spain, but they are very heterogeneous among themselves, but in general they do not usually have a specific admission criteria criteria, except for age, and their objective to offer new knowledge, creating social media networks and participating in intergenerational relationships. The purpose is not to obtain a university degree, but rather to improve the quality of life of the elderly (Sitges, 2019).

The second group (institutionalized subjects) were recruited from multiple residential centers in the Autonomous Communities of Castilla, León and Valencia (Spain), in order to maintain the same contextual characteristics as the first group. The justification for using a dichotomized sample in autonomous and institutionalized older adults is based on the objective of offering a broader insight of the types of reserves evaluated, since in previous works they had been carried out in one condition or another. Since environmental enrichment is key to cognitive reserve, variations in the two habitat conditions are relevant to discriminate (Adam et al., 2013; Chapko, 2018; Lavrencic et al., 2018). In addition, due to the differential characteristics of the lifestyles in both contexts, a large difference can be predicted in the physical activity variable, a relevant factor in cognitive reserve, as has been pointed out (Cheng, 2016). We must also take note of the differential role in terms of life project and performance of significant life activities that both contexts enable and reinforce (Pettigrew and Soldan, 2019).

Secondly, the battery of tests was applied. The administration of these tests was carried out individually, lasting approximately an hour and a quarter with each subject. The evaluation period was carried out between the months of March and October, and in all cases informed consent for participation was previously collected. In addition, this study has strictly complied with the ethical criteria indicated in the Declaration of Helsinki (revised in 2013) for research of this type. Finally, as inclusion criteria, in addition to accepting the indicated conditions (voluntariness to participate, present legal authorization and waiver of remuneration), the participants could not present any cognitive impairment. Failure to meet any of the above criteria were grounds for exclusion.

### Data analysis

We built mediation analyses testing each possible mediator of cognitive, physical and motivational reserve on cognitive scores (MoCA) as the dependent variable. The predictors were age, educational level, sex, and institutionalization. Cognitive reserve was analyzed using both CRQ, and CRS scores. Physical reserve was analyzed with IPAQ scores, and motivational reserve was analyzed with PIL scores. For each predictor and mediator, we present unstandardized scores, and *p* values using the Sobel test for mediation analyzed in the Lavaan package in R.

The mediation analysis is presented using sociodemographic variables of age, sex, educational level and institutionalization as predictors. Cognitive reserve was assessed using the Cognitive Reserve Questionnaire and the Cognitive Reserve Scale, for the physical reserve the International Physical Activity Questionnaire was used, in which previous studies have found relevance in cognitive aging. The indirect motivational reserve was assessed using the Purpose in Life Questionnaire, and the cognitive performance was assessed with the MoCA test. If the indirect effect (column “a*b” in Tables 1–4) had greater weight than the direct effect (column “c”), this would mean that motivational reserve is an important mediating factor, beyond the mentioned variables.

**Table 1:** Mediation analysis of the four sociodemographic predictors of cognitive performance (assessed with MoCA and M@T) contrasted with cognitive reserve as a mediating variable (assessed with CRQ and CRS).

	**Indirect effect**				**Direct effect**	**Total effect**
	a	b	a*b	Prop.	c	(a*b) + c
**CRQ→MoCA**						
Age	−0.201***	0.495***	−0.100***	0.47	−0.111***	−0.211***
Education	4.084***	0.677***	2.764***	1.12	−0.300	2.465***
Sex (Women)	−0.485	0.625 ***	−0.303	0.27	−0.832*	−1.134*
Institutionalization	−5.973***	0.477***	−2.847***	0.57	−2.153***	−5.000***
**CRS→MoCA**						
Age	−0.593***	0.127***	−0.075***	0.36	−0.136***	−0.211***
Education	8.465***	0.129***	1.093***	0.44	1.371***	2.465***
Sex (Women)	2.709	0.185***	0.501	0.44	−1.636***	−1.134*
Institutionalization	−19.26***	0.104***	−2.006***	0.40	−2.994***	−5.000***
**CRQ→M@T**						
Age	−0.201***	0.930***	−0.187***	0.40	−0.282***	−0.470***
Education	4.084***	1.209***	4.937***	0.93	0.364	5.300***
Sex (Women)	−0.485	1.265***	−0.613	0.51	−0.587	−1.200
Institutionalization	−5.973***	0.752***	−4.495***	0.38	−7.298***	−11.79 3***
**CRS→M@T**						
Age	−0.593***	0.258***	−0.153***	0.33	−0.317***	−0.470***
Education	8.453***	0.269***	2.276***	0.43	3.024***	5.300***
Sex (Women)	2.744	0.389***	1.067	0.89	−2.268**	−1.200
Institutionalization	−19.278***	0.162***	−3.120***	0.26	−8.673***	−11.79 3***

*p < 0.05, **p < 0.01, ***p < 0.001. The indirect effect is evaluated from the effect of the predictor on the mediating variable (a), and in turn from the mediating variable on cognitive performance (b). The indirect effect is reported by multiplying the two previous coefficients (a*b). The direct effect is the coefficient obtained directly from each predictor on the cognitive performance score (c).

**Table 2:** Mediation analysis of the four sociodemographic predictors of cognitive performance (assessed with the MoCA and M@T) contrasted with physical reserve as a mediating variable (assessed with the IPAQ).

	**Indirect effect**				**Direct effect**	**Total effect**
	a	b	a*b	Prop.	c	(a*b) + c
**MoCA**						
Age	0.014***	−1.294***	−0.018**	0.09	−0.193***	−0.211***
Education	−0.286***	−0.975**	0.279**	0.11	2.186***	2.465***
Sex (Women)	0.098	−1.811***	−0.177	0.16	−0.957	−1.134*
Institutionalization	0.440***	−1.012***	−0.445**	0.09	−4.555***	−5.000***
**M@T**						
Age	0.014***	−2.652***	−0.036**	0.08	−0.434***	−0.470***
Education	−0.286***	−2.005***	0.574**	0.11	4.726***	5.300***
Sex (Women)	0.098	−3.851***	−0.376	0.31	−0.824	−1.200
Institutionalization	0.440***	−1.881***	−0.828**	0.07	−10.966***	−11.80***

*p < 0.05, **p < 0.01, ***p < 0.001. The indirect effect is evaluated based on the effect of the predictor on the mediating variable (a), and in turn on the mediating variable on performance in cognitive performance (b). The indirect effect is reported by multiplying the two previous coefficients (a*b). The direct effect presented is the coefficient obtained directly from each predictor on cognitive performance (c).

**Table 3:** Mediation analysis of the four sociodemographic predictors of cognitive performance (assessed with the MoCA and M@T) contrasted with motivational reserve as a mediating variable (assessed with the PIL).

	Indirect effect				Direct effect	Total effect
	a	b	a*b	Prop.	c	(a*b) + c
**MoCA**						
Age	0.083	0.027*	0.002	<0.01	−0.213***	−0.211***
Education	2.581*	0.005	0.014	<0.01	2.451***	2.465***
Sex (Women)	−0.420	0.020	−0.009	<0.01	−1.126*	−1.134*
Institutionalization	−1.400	0.016	−0.022	<0.01	−4.978***	−5.000***
**M@T**						
Age	0.083	0.052**	0.004	<0.01	−0.474***	−0.470***
Education	2.581*	0.005	0.012	<0.01	5.288***	5.300***
Sex (Women)	−0.420	0.037	−0.016	0.01	−1.184	−1.200
Institutionalization	−1.400	0.026	−0.036	<0.01	−11.757***	−11.80***

*p < 0.05, **p < 0.01, ***p < 0.001. The indirect effect is evaluated based on the effect of the predictor on the mediating variable (a), and in turn on the mediating variable on performance (b). The indirect effect is reported by multiplying the two previous coefficients (a*b). The direct effect presented is the coefficient obtained directly from each predictor on cognitive performance (c).

**Table 4:** Mediation analysis of sociodemographic predictors of cognitive performance (assessed with MoCA and M@T) contrasted with cognitive reserve as a mediating variable (assessed with PIL), separating the effects in the samples of institutionalized and non-institutionalized participants.

**Institutionalized**	**Indirect effect**				**Direct effect**	**Total effect**
	a	b	a*b	Prop.	c	(a*b) + c
**Yes – MoCA**						
Age	0.200	0.012	0.002	0.01	−0.136***	−0.134***
Education	1.588	0.002	0.003	<0.01	1.957***	1.960***
Sex (Women)	3.190	0.012	0.038	0.01	−2.615***	−2.577***
**No – MoCA**						
Age	0.507*	0.027**	0.014	0.44	−0.046	−0.032
Education	4.073*	0.017	0.067	0.06	1.000***	1.067***
Sex (Women)	−4.999	0.024*	−0.120	0.30	−0.281	−0.401
**Yes – M@T**						
Age	0.200	0.050	0.010	0.04	−0.270***	−0.260***
Education	1.588	0.027	0.043	<0.01	4.389***	4.433***
Sex (Women)	3.190	0.047	0.149	0.04	−4.123**	−3.974**
**No – M@T**						
Age	0.507*	0.011	0.006	0.60	0.004	0.010
Education	4.073*	−0.000	−0.001	<0.01	1.407***	1.407***
Sex (Women)	−4.999	0.011	−0.055	0.27	−0.146	−0.201

*p < 0.05, **p < 0.01, ***p < 0.001. The indirect effect is evaluated based on the effect of the predictor on the mediating variable (a), and in turn on the mediating variable on cognitive performance.

(b). The indirect effect is reported by multiplying the two previous coefficients (a*b). The direct effect presented is the coefficient obtained directly from each predictor on cognitive performance (c).

Figure 1 shows the structure of the mediations that were analyzed. We used the convention of testing the significance of the direct effect (c), and of the indirect effect (a*b), and we considered that there was an effect of the mediator over the dependent variable when the indirect effect was statistically significant (*p* < 0.05). Therefore, we considered both complete and partial mediations of the cognitive (CRQ, CRS scores), physical (IPAQ scores) and motivational (PIL scores) reserves.

## Results

### Cognitive reserve

The summary of the direct, indirect, and total effects of age, educational level, sex and institutionalization on cognition, with cognitive reserve as mediator, is presented in Table 1. All four sociodemographic variables had statistically significant total effects, which indicates that they were predictors of cognition measured by MoCA and M@T.

In the case of age, there was a significant and negative mediation effect of cognitive reserve, with both Cognitive Reserve Questionnaire and Cognitive Reserve Scale scores, on cognition. The direct effect remained significant and was negative, indicating that overall, age had a negative correlation with cognition, whereas age increased, MoCA/M@T scores decreased. Looking at the indirect effect, the relation between age and Cognitive Reserve Questionnaire and Cognitive Reserve Scale scores was negative, which explains why, even though cognitive reserve has a positive relation with cognition, the overall effect of age was still negative. The mediation proportion was between a third (CRS scores) and a half (CRQ scores) of the total effect.

In the case of educational level, there was a significant and positive mediation effect of cognitive reserve with both Cognitive Reserve Questionnaire and Cognitive Reserve Scale scores. Interestingly, the direct effect remained significant only in the CRS scores. With CRQ scores, the mediation proportion was even larger than a 100% of the total effect. The effects of educational level on cognitive reserve (both CRQ and CRS scores), and of cognitive reserve on cognition, were positive, therefore, the overall indirect effect was also positive, indicating that the higher educational level, the higher the MoCA scores.

Sex had no significant indirect effect through cognitive reserve on cognition, with either Cognitive Reserve Questionnaire and Cognitive Reserve Scale scores. Nevertheless, there was a statistically significant positive effect of cognitive reserve on cognition, after controlling for the direct effect of sex. These results indicate that cognitive reserve did not depend on the sex of the participants, but cognition did varied with cognitive reserve levels.

Institutionalization showed a similar pattern as age, but these indirect effects are more difficult to interpret, given that institutionalized older adults had higher ages. The mediation proportion was a bit higher than that of age, 40% (CRS scores) and 57% (CRQ scores) for each type of measure of cognitive reserve.

Overall, cognitive reserve mediated the effects of age, educational level and institutionalization on cognition. In all cases, cognitive reserve had a positive effect on cognition, which was summed to the negative effect of age and institutionalization, and the positive effect of educational level.

### Physical reserve

The summary of the direct, indirect, and total effects of age, educational level, sex, and institutionalization on cognition, with physical reserve as mediator, is presented in Table 2. Age, educational level and institutionalization, but not sex, showed statistically significant total effects, which indicates that they were predictors of cognition measured by MoCA and M@T.

In the case of age, there was a significant and negative mediation effect of physical reserve, measured with IPAQ, on cognition. The direct effect remained significant and was negative, indicating that overall, age had a negative correlation with cognition, as age increased, MoCA/M@T scores decreased. Parsing the indirect effect, the relation between age and IPAQ scores was positive, indicating that as age increased, physical activity decreased too (higher IPAQ scores mean lower physical activity). Further, the effect of IPAQ on MoCA and M@T scores was negative, indicating that as physical activity increased, MoCA scores increased. The overall indirect effect was negative, but small, the mediation proportion was less than 10% of the total effect.

Educational level showed a significant positive indirect effect on cognition, and the direct effect remained significant and positive. Indicating that, overall, educational level had positive effects on cognition, either directly, or indirectly *via* physical activity. This indirect effect is constructed with a negative effect of educational level on IPAQ scores, which means that higher educational level is associated with higher physical activity, and a negative effect of IPAQ scores on MoCA/M@T scores, which is interpreted as an association of higher physical activity with higher MoCA/M@T scores. The mediation proportion is similar to that of age, of 11% of the total effect. Overall, higher educational level was associated with higher cognitive scores, either *via* a direct effect and *via* an indirect effect through higher physical activity.

Even though sex did show a direct effect only for MoCA scores, it did not show an indirect effect through physical activity, but there was a statistically significant effect of physical activity on cognition. The effect of IPAQ scores on MoCA scores was negative, indicating that higher physical activity was associated with higher cognitive scores.

Institutionalization showed both significant direct and indirect effects, and the mediation proportion was similar to that of age, which probably indicates that these indirect effects were driven mostly by the age difference among groups.

In summary, physical reserve mediated the effects of age, educational level and institutionalization on cognition. The mediation proportions of physical reserve were lower than those for cognitive reserve. The pattern of results showed that age and institutionalization had negative direct and indirect effects overall, but educational level showed positive direct and indirect effects on cognition. Because of the way the Physical Activity Questionnaire scores are interpreted, where lower scores indicate higher physical activity, the effects of Physical Activity Questionnaire on MoCA and M@T scores were all negative.

### Motivational reserve

The summary of the direct, indirect, and total effects of age, educational level, sex, and institutionalization on cognition, mediated by motivational reserve measured with the PIL test, is presented in Table 3. All four sociodemographic variables showed statistically significant total effects, which indicates that they were predictors of cognition measured by MoCA and M@T, except for sex as the independent variable for M@T scores. In neither case of age, educational level, sex, and institutionalization, there were significant indirect effects through PIL scores. The mediation proportion in either case was less than 1% of the total effects. In the mediation analyses with age as the predictor, PIL scores showed statistically significant positive effects on MoCA scores, but the *p* value was near threshold (*p* = 0.012), which should be interpreted with caution. Also, educational level showed a statistically significant effect on PIL scores, with the same caveat about the p value (*p* = 0.028).

Given the absent indirect effects *via* motivational reserve indexed with PIL scores, we decided to explore separating institutionalized and non-institutionalized older adults, and run the same analyses in both groups only for the motivational reserve. The summary of that analysis is shown in Table 4.

In the case of institutionalized older adults, neither age, educational level, or sex, showed significant indirect effects through motivational reserve on cognition. The mediation proportions were less than 1%. Interestingly, in the case of non-institutionalized older adults, age showed a statistically significant effect on PIL scores, and PIL scores showed an effect on MoCA scores, but the indirect effect overall did not reach significance (*p* = 0.098).

In the non-institutionalized sample there were no statistically significant indirect effects of either age, educational level or sex on cognition, *via* PIL scores. Though, educational level showed a statistically significant effect on PIL scores (*p* = 0.028), and PIL scores an effect on MoCA scores (*p* = 0.011), with sex as the predictor, but these effects were fairly low, even though they lowered the direct effects of age and sex on cognition, rendering them non-statistically significant.

In summary, motivational reserves measured with the PIL test did not show a mediation effect on cognition over age, educational level, sex, or institutionalization. When looking at the sample of non-institutionalized older adults, PIL scores showed significant effects in relation to age, although the overall indirect effect was not significant.

## Discussion

The aim of this study was to examine the influence of cognitive reserve, physical reserve and motivational reserve on the cognitive state of the participants and to analyze the independent variables.

With regards to the cognitive reserve and the age variable, it is worth mentioning that the results found in this study coincide with the results from other authors, like Brichko et al. (2022), who analyzed the role that age has on the level of cognitive reserve and white matter in the elderly. This study concluded that cognitive reserve should be considered as a compensatory variable among older adults. Other studies developed by Ekdahl et al. (2022) and Barulli et al. (2022), in which they used subjects who had been studied through neuropsychological evaluations and structural neuroimaging. The objective was to analyze the associations between these data and the strategic flexibility measures. The results indicated that age had a negative effect on strategic flexibility, but those with higher cognitive reserve had a better cognitive flexibility. These results are consistent with those obtained in this research.

Cuéllar et al. (2022) have carried out a study at the Anahuac University of Mexico in which they have analyzed the role of personality in the cognitive reserve. Various factors have been found to contribute to the formation of cognitive reserves. They found differences in the neuroticism variable between men and women, and less neuroticism, less extraversion and greater conscientiousness at older ages. Thus, they concluded that there are predictors of cognitive reserve in people over the age of 18.

Regarding the sex variable, no significant differences were observed in this study, but it should be noted that, in a recent study, differences were found in favor of men in the education level, languages, occupation and total score, since it was measured by the Cognitive Reserve Questionnaire. These authors also analyzed the education level of the subjects, suggesting that education could stimulate the search for intellectual enrichment and that, in turn, this would improve the cognitive and functional performance of older people Grasso et al. (2021). On the other hand, a recent study has researched the effect of maintenance and reserve factors measured through neuronal efficiency called phase specificity, in which they concluded that women obtain better results, meaning they have a better and higher brain performance (Argiris et al., 2022).

Another variable studied is the educational level in which in previous studies done by the same authors (Wobbeking et al., 2020) they reached the same conclusion in this present study, stating that the educational level is one of the strongest predictors of cognitive reserve. It should be noted that the Cognitive Reserve Questionnaire scores include questions about the educational level, but the CRS scores do not. This explains why the indirect effect of the educational level through the CRQ scores explained the entire effect and the direct effect disappeared. Therefore, it would be advisable to use CRS to measure different variables, complementary to the educational level.

The results are in line with other studies that support the hypothesis that cognitive reserve influences the delay in the development of cognitive impairment symptoms. In a recent study done by Ihle et al. (2022), with a Portuguese population, it is evident that healthy cognitive functioning is mediated by cognitive reserve (measured with indicators of educational levels and types of professions) and perceived quality of life. This reinforces the important role of cognitive reserve in maintaining healthy aging, especially in its health-related quality of life (HRQoL) dimension.

The second variable studied in this research is the physical reserve. The results show the positive association with cognitive function and the improvement of the motivational reserve, results that coincide with the scientific literature. A study conducted by Mejía et al. (2020) has analyzed the relation between the practice of physical activity and emotional state in adults aged 60 to 70 years, concluding that people who perform low physical activity present a negative emotional state characterized by the presence of symptoms such as anxiety and anger.

Motivational reserve on non-institutionalized older adults, effect of age on PIL, and of PIL on MoCA were significant, but not the full indirect effect. If there were an indirect effect, which we failed to detect due to insufficient statistical power, the effect could be positive, but small, where higher age would be associated with higher purpose in life, and further higher purpose in life would be associated with higher cognitive scores. These results are novel since they relate the cognitive reserve to motivational reserve and specifically to the meaning of life. They extend and converge with many studies that relate the meaning of life with positive neuropsychological aspects such as less anxiety and depression, greater physical, psychological and social well-being, as well as greater life expectancy and optimism (Park et al., 2004; Peterson and Seligman, 2004; Kim et al., 2013; Proyer et al., 2013; Niemiec, 2014; Zilioli et al., 2015; Cohen et al., 2016; Steptoe, 2019; Steptoe and Fancourt, 2019; Trudel-Fitzgerald et al., 2019; Kim et al., 2020; Weziak-Bialowolska et al., 2021; Kim et al., 2022).All these variables can be related by key factors such as positive emotions, which are directly related to these constructs.

In this study, the mediating effects of cognitive reserve, physical reserve and motivational reserve on the cognitive performance of older people were reviewed, based on the predictor variables of age, educational level and institutionalization. The cognitive reserve (assessed with CRQ and CRS) and the physical reserve (assessed with the IPAQ) were found to have important partial mediating effects over age, educational level, and institutionalization over cognitive performance (assessed with MoCA and M@T). The motivational reserve (assessed with the PIL test), had smaller effects but of interest to be reviewed in future studies, especially in the non-institutionalized older population. This study reflects the important relation between cognitive impairment and cognitive reserve, physical reserve and motivational reserve, these being key factors in the process of preventing pathological aging.

We have to recognize two types of limitations in this study. The first is related to the type of methodology used, since it is a cross-sectional study and has a more limited explanatory power than that which would have been made with a longitudinal monitoring of the sample. And the second limitation would be circumscribed to the instruments used, especially regarding the measurement of cognitive reserve. This is a matter of controversy in the literature, given the difference that exists between the measurements scales of the constructs. Although we must point out that the one used meets the two basic criteria required in most works (Kartschmit et al., 2019): include the relevant key aspects (education-training, profession-education, physical activities-leisure) and being among the six instruments with better psychometric properties.

## Data availability statement

The raw data supporting the conclusions of this article will be made available by the authors, without undue reservation.

## Ethics statement

Ethical review and approval were not required for the study on human participants in accordance with the local legislation and institutional requirements. The patients/participants provided their written informed consent to participate in this study.

## Author contributions

AC, MWS, and BB-L: conceptualization and resources. MM-R and JU-L: methodology and data curation. MWS: validation. MM-R: formal analysis. MWS, BB-L, and AC: investigation. EC-R: writing—original draft preparation. EC-R and MM-R: writing—review and editing. MWS, JU-L, AC, and BB-L: visualization and project administration. AC, MM-R, and MWS: supervision. All authors contributed to the article and approved the submitted version.

## Conflict of interest

The authors declare that the research was conducted in the absence of any commercial or financial relationships that could be construed as a potential conflict of interest.

The reviewer JL declared a past collaboration with the authors AS and BB-L to the handling editor.

## Publisher’s note

All claims expressed in this article are solely those of the authors and do not necessarily represent those of their affiliated organizations, or those of the publisher, the editors and the reviewers. Any product that may be evaluated in this article, or claim that may be made by its manufacturer, is not guaranteed or endorsed by the publisher.
